# Exploring the Role of Immune Cells in Glioma: Causal Associations and Clinical Implications

**DOI:** 10.7150/ijms.116560

**Published:** 2025-06-12

**Authors:** Yimin Pan, Xiaoshun Sun, Fushu Luo, Zheng Chen, Chunbo Liu, Yongye Zhu, Jiaxiang Wang, Ruixuan Chen, Qing Liu, Changwu Wu, Jun Tan

**Affiliations:** 1 Department of Neurosurgery, Xiangya Hospital, Central South University, Changsha, Hunan, 410008, China.; 2 National Clinical Research Center for Geriatric Disorders, Xiangya Hospital, Central South University, Changsha, Hunan, 410008, China.; 3 Clinical Research Center for Skull Base Surgery and Neuro-oncology in Hunan Province, Changsha, Hunan, 410008, China.

**Keywords:** immune cell, glioma, Mendelian randomization, prognosis, drug sensitivity

## Abstract

**Background:** Gliomas, the predominant malignant neoplasm of the central nervous system, are notorious for their recurrence and unfavourable prognosis. Immune cells play a pivotal role in the progression of various solid tumors, including gliomas. This study aims to explore the potential causal effect of immune cells on the risk of glioma and the association between immune cells and clinical characteristics in glioma.

**Materials and Methods:** This study used the public genome-wide association studies (GWAS) summary data of 731 immune cell traits and gliomas to perform two-sample Mendelian randomization (MR) analysis. The MR analysis primarily employed the inverse variance weighting (IVW) method, supplemented by three additional methods, alongside comprehensive pleiotropy and heterogeneity analyses. In addition, 151 glioma samples were collected for RNA-Seq to construct the CSUXY cohort, and RNA-Seq data and clinical information of 588 glioma samples in the TCGA cohort were collected. The associations between immune cell abundance and clinical characteristics and drug sensitivity of each sample were inferred in the two cohorts.

**Results:** Based on the IVW method, this study identified potential causal associations between 16 immune cell traits and the risk of glioma. The other three MR analysis methods had consistent causal directions with the IVW method and there was no horizontal pleiotropy and heterogeneity. Higher levels of immune cell infiltration were observed in IDH wild-type and 1p19q non-codel gliomas compared to IDH mutant and 1p19q codel gliomas across both the CSUXY and TCGA cohorts. In addition, the abundance of immune cells was also associated with the grade, histological subtype and prognosis of gliomas. Finally, this study also identified broad associations between immune cell abundance and drug sensitivity in glioma.

**Conclusion:** This study supports the causal effects of specific immune cell traits on glioma and confirms the associations between immune cells and clinical characteristics, as well as drug sensitivity in glioma, providing evidence for the development of immune cell-based biomarkers.

## Introduction

Glioma is a neuroepithelial tumor and the most common primary malignant tumor in the central nervous system. Its main characteristics include poor prognosis, easy recurrence and resistance to a variety of treatment modalities. The median survival of high-grade gliomas is usually only 12 to 15 months, while the 5-year survival rate is less than 5% 1,2. Despite a multi-modality treatment strategy comprising surgery, chemotherapy, radiation and/or immunotherapy, the outcomes still remain dismal 3. Therefore, finding new therapeutic strategies to meet these challenges has become the core task of glioma research. Further exploration of the pathogenesis factors and potential biomarkers of glioma will contribute to the development of new therapeutic strategies.

Recent advancements in tumor immunology highlight the significant role of immune cells as key components of the tumor microenvironment (TME), playing a crucial part in regulating tumor progression 4. The interactions between these immune cells and tumor cells can either inhibit or promote the development of gliomas. This understanding is essential for advancing the development of immunotherapeutic agents. Immune cells have a complex role in tumorigenesis; they can suppress tumor growth by eliminating cancer cells, but they may also facilitate tumor progression by providing growth and survival factors 4,5.

For example, tumor-associated macrophages (TAMs) represent the most abundant immune cell population within the TME of glioma, accounting for approximately 50% of the TME cells in gliomas 6,7. These macrophages respond to various factors secreted by cancer cells, releasing a range of growth factors and cytokines, which play a crucial role in promoting tumor development within the TME. Studies have demonstrated that disrupting the function of microglia and macrophages in mouse models of gliomas significantly inhibits tumor proliferation 8. Furthermore, the high infiltration rate of macrophages in gliomas is closely associated with poor prognosis 9, a pattern that mirrors observations in other tumor types 10.

In contrast, dendritic cells (DCs) have the capability to recognize tumor antigens and transport them to tumor-draining deep cervical lymph nodes, thereby triggering T cell-mediated immune responses 11-13. Furthermore, dendritic cells can produce chemokines that recruit cytotoxic T lymphocytes into the TME, effectively inhibiting the progression of gliomas 14,15. Despite significant advancements in the study of immune cells, the relationship between immune cell traits and gliomas remains inconsistent. It is essential to further investigate the causal relationships and clinical implications between immune cell traits and glioma.

Mendelian randomization (MR) leverages genetic variants as instrumental variables (IVs) to infer causal relationships between exposures (e.g., environmental factors, lifestyle choices, or immune cell traits) and glioma outcomes 16,17. Unlike observational studies, which can be confounded by biases such as measurement errors or external variables, MR uses genetic variants that are randomly assigned at conception, minimizing confounding and providing more reliable results 18,19. Additionally, MR is less susceptible to measurement errors, as genetic data is objective and precise compared to self-reported data, which can be inaccurate 20.

Although randomized controlled trials (RCTs) are considered the gold standard for establishing causal effects, their practical implementation can be challenging 21. MR serves as an alternative to RCTs by leveraging summary data from genome-wide association studies (GWAS) and employing single nucleotide polymorphisms (SNPs) to facilitate causal inference 19. This method enhances the reliability of epidemiological research, making it less susceptible to biases and providing more robust insights into the relationships between various health-related variables 16,17. Therefore, it is worthwhile to use MR methods to infer the potential causal relationship between immune cell traits and glioma.

In addition, the rise of transcriptomic sequencing has promoted the inference of immune cell infiltration levels based on sequencing data. Previous studies have also confirmed the reliability of estimating immune cell infiltration from RNA sequencing (RNA-Seq) data 22,23. In this study, we combined MR methods based on GWAS data and RNA-Seq data of a large number of glioma samples to explore the causal and clinical associations between immune cells and glioma. This study aims to further reveal the potential pathogenic factors and biomarkers of glioma.

## Methods

### Data source

The GWAS summary data for immune cells used in this study were derived from the study by Orrù *et al.* 24 and can be downloaded from IEU Open GWAS (https://gwas.mrcieu.ac.uk/) with the accession numbers ebi-a-GCST90001391 to ebi-a-GCST90002121. The GWAS cohort covered 731 broad immune traits from 3,757 Sardinians, including absolute cell counts, median fluorescence intensities of surface antigens, morphological parameters, and relative cell counts. The GWAS summary data of human gliomas was obtained from the FinnGen database (https://www.finngen.fi/en) 25, and this GWAS cohort included glioblastoma and astrocytoma. The data sources and characteristics of immune cell and glioma GWAS cohorts were summarized in Supplementary Table S1. For transcriptomic analysis, we collected 151 glioma samples from Xiangya Hospital, Central South University for RNA-Seq, and complete follow-up information was collected for all samples. The collection of human tissues was approved by the Medical Ethics Committee of Xiangya Hospital of Central South University (Approval number: 202401003) and written informed consent was provided by all of the patients. This cohort was named CSUXY, and the clinical characteristics of this cohort were summarized in Supplementary Table S2. We downloaded normalized RNA-Seq data and clinical information of 588 glioma samples from The Cancer Genome Atlas (TCGA) from UCSC Xena (https://xena.ucsc.edu/). All included patients had primary tumors, and the samples were sourced from their initial surgeries. In addition, we previously collected nine fresh glioma samples from the Department of Neurosurgery, Xiangya Hospital for single-cell RNA sequencing (scRNA-seq). Detailed information about the nine samples can be found in the previous study 26.

### Instrumental variable selection

In this study, SNPs were selected as IVs. Although the conventional threshold for IVs is *P* < 5 × 10^-8^ to minimize the risk of false positives, this approach can be problematic in certain situations. For instance, when the number of IVs exceeding this threshold is small, the analysis may be inadequate, or in some unbiased screening situations, the results may be exaggerated 27. The source literature for immune cell GWAS data adopted a more lenient threshold of *P* < 1 × 10^-5^ 24, which may increase the risk of false positives. After comprehensively considering the risk of false positives and the number of IVs, we adopted a genome-wide significance threshold of *P* < 5 × 10^-6^ for the selection of IVs related to immune cell traits. Ye *et al.* also used this threshold to screen IVs for immune cell traits 28. To mitigate linkage disequilibrium among the IVs, we applied a clumping distance of 10,000 kb and an R2 threshold of < 0.001 during the clumping process to evaluate the SNPs. Furthermore, to circumvent the issue of weak instrument bias, we excluded SNPs with F statistics < 10 from the analysis. Careful harmonization of the SNPs between the exposure and outcome variables was ensured, guaranteeing that they corresponded to the same alleles. Additionally, we also eliminated SNPs with a close association with glioma (*P* < 5 × 10^-5^) and palindromic SNPs. To rule out the possibility of reverse causal associations, we conducted the Steiger test, and we subsequently excluded SNPs that failed to meet the test criteria.

### RNA-seq of glioma samples

RNA-Seq was performed as described previously 26,29,30. Total RNA was extracted from tissue samples using TRIzol® Reagent following the manufacturer's instructions. RNA quality and quantity were rigorously assessed using the Agilent 5300 Bioanalyzer and ND-2000 NanoDrop. Only high-quality RNA samples meeting strict criteria were used for downstream processing: OD260/280 ratio of 1.8-2.2, OD260/230 ratio ≥ 2.0, RNA Integrity Number (RIN) ≥ 6.5, 28S:18S ribosomal RNA ratio ≥ 1.0, and total RNA quantity > 1 μg. For library preparation, 1 µg of total RNA was used to construct the RNA-seq transcriptome library using the Illumina® Stranded mRNA Prep, Ligation kit. Messenger RNA (mRNA) was isolated via polyA selection using oligo(dT) beads, followed by fragmentation to generate fragments of approximately 300 bp. Double-stranded cDNA was synthesized using a SuperScript double-stranded cDNA synthesis kit (Invitrogen, CA) with random hexamer primers. The cDNA underwent end-repair, phosphorylation, and 'A' base addition to prepare for adapter ligation. Libraries were size-selected using 2% Low Range Ultra Agarose to enrich for fragments of ~300 bp and amplified by PCR with Phusion DNA polymerase (NEB) for 15 cycles. The final libraries were quantified using Qubit 4.0 and validated for size distribution using the Agilent Bioanalyzer. Paired-end sequencing (2 × 150 bp) was performed on the Illumina NovaSeq 6000 platform. Raw sequencing reads were subjected to stringent quality control using fastp. Adapter sequences and reads without insert fragments were removed, and low-quality bases (quality score < 20) were trimmed from the 3' end of reads. Reads with remaining bases having quality scores < 10 or an N (ambiguous base) ratio exceeding 10% were discarded. Additionally, reads shorter than 20 bp after trimming were excluded. These steps produced high-quality clean reads for downstream analysis. Quality metrics, including base composition distribution, base quality distribution, and base error rate distribution, were evaluated to confirm the reliability of the sequencing data. Clean reads were aligned to the reference genome in orientation-aware mode using HISAT2. The alignment results were assessed for sequencing saturation, gene coverage, and distribution of reads across genomic regions and chromosomes. Transcriptome assembly was performed using StringTie in a reference-based approach, which reconstructed transcript structures and quantified their abundances. Gene and transcript expression levels were quantified using RSEM (version 1.3.3) based on the number of reads mapped to genomic regions. Expression levels were normalized to transcripts per million (TPM) to account for differences in sequencing depth and transcript length across samples, enabling accurate cross-sample comparisons.

### scRNA-seq and deconvolution analysis

scRNA-seq was performed on nine glioma samples using the droplet-based 10× Genomics platform (10x Genomics, Pleasanton, CA, USA). The detailed quality control process and analysis methods of scRNA-seq can be found in our previous published research 26. Briefly, after filtering, a total of 94629 high quality cells were retained for subsequent analysis based on “Seurat” package. The Harmony algorithm was used to remove batch effects between samples. Classic cell markers were used to identify the cell type of each cluster. The BayesPrism deconvolution method was used to infer the cellular composition of the CSUXY and TCGA cohorts based on the expression matrix of the scRNA analysis according to default parameters 31.

### MR analysis

MR relies on three core assumptions to ensure valid causal inference: (1) the genetic variants used as instrumental variables (IVs) must be strongly associated with the exposure of interest (relevance assumption); (2) the genetic variants should not be associated with any confounding factors that affect the exposure-outcome relationship (independence assumption); and (3) the genetic variants must influence the outcome only through the exposure of interest, with no direct or alternative pathways (exclusion restriction assumption). To explore the causal relationship between immune cell traits and glioma, we employed four different methods for MR analysis. These methods included inverse variance-weighted (IVW) 32, MR-Egger regression 33, weighted median 34, and weighted mode 35. A comparative study previously demonstrated the superior power of the IVW method under specific conditions 34. In light of this, our study primarily focuses on the findings obtained using the IVW method, while considering the results from the other three methods as supplementary information. To address potential biases, especially horizontal pleiotropy, we implemented several robust methods and sensitivity analyses. First, we performed MR-Egger regression, which provides an estimate of causal effect that is less sensitive to pleiotropy by allowing for an intercept term that captures directional pleiotropy. Horizontal pleiotropy was assessed using the MR-Egger intercept, where a *p*_-intercept_ < 0.05 indicated the presence of horizontal pleiotropy. Heterogeneity among the included single SNPs in each analysis was evaluated using Cochran's Q test in IVW and MR-Egger methods, with a significance level of *p* < 0.05 indicating high heterogeneity. In addition, we conducted leave-one-out analyses to identify and exclude potential outlier SNPs that might disproportionately influence the results due to pleiotropic effects. All MR analyses were performed using the “TwoSampleMR” (version 0.5.7) and “MendelianRandomization” (version 0.8.0) packages. A suggestive causal association was defined as a *p* < 0.05.

### Immune infiltration analysis

We performed immune infiltration analysis according to the methods used in previous studies 23,36-38. Specifically, we collected a gene set of 28 immune cells from the study of Charoentong *et al.* 39, and then we quantified the relative level of immune cell infiltration in each sample using the single-sample gene set enrichment analysis (ssGSEA) method based on the R package “GSVA”. In addition, we used the quanTIseq algorithm to quantify the absolute proportions of the 10 immune cell types in each sample 40.

### Drug sensitivity analysis

As previously described 41, we obtained the gene expression data of 809 tumor cell lines and corresponding response data for each cell line from the Genomics of Drug Sensitivity in Cancer (GDSC) database. The data was normalized and converted to IC_50_ values. Subsequently, the IC_50_ values for each drug were estimated for individual glioma patients using the oncoPredict algorithm 42 based on the gene expression profiles of cell lines and the drug response data.

### Statistical analysis

Statistical comparisons between two groups were conducted using either an unpaired Student's t-test or a Wilcoxon rank sum test. On the other hand, when comparing differences among more than two groups, either a one-way ANOVA or a Kruskal-Wallis test was employed. Univariate Cox regression was used to assess the prognostic significance of individual immune cells. Spearman's correlation analysis was used to quantify the correlation between two groups. All statistical calculations were performed using R software (version 4.3.1) and *p* < 0.05 was regarded as statistically significant.

## Results

### Causal effects of immune cell traits on the risk of glioma

**Figure 1:**
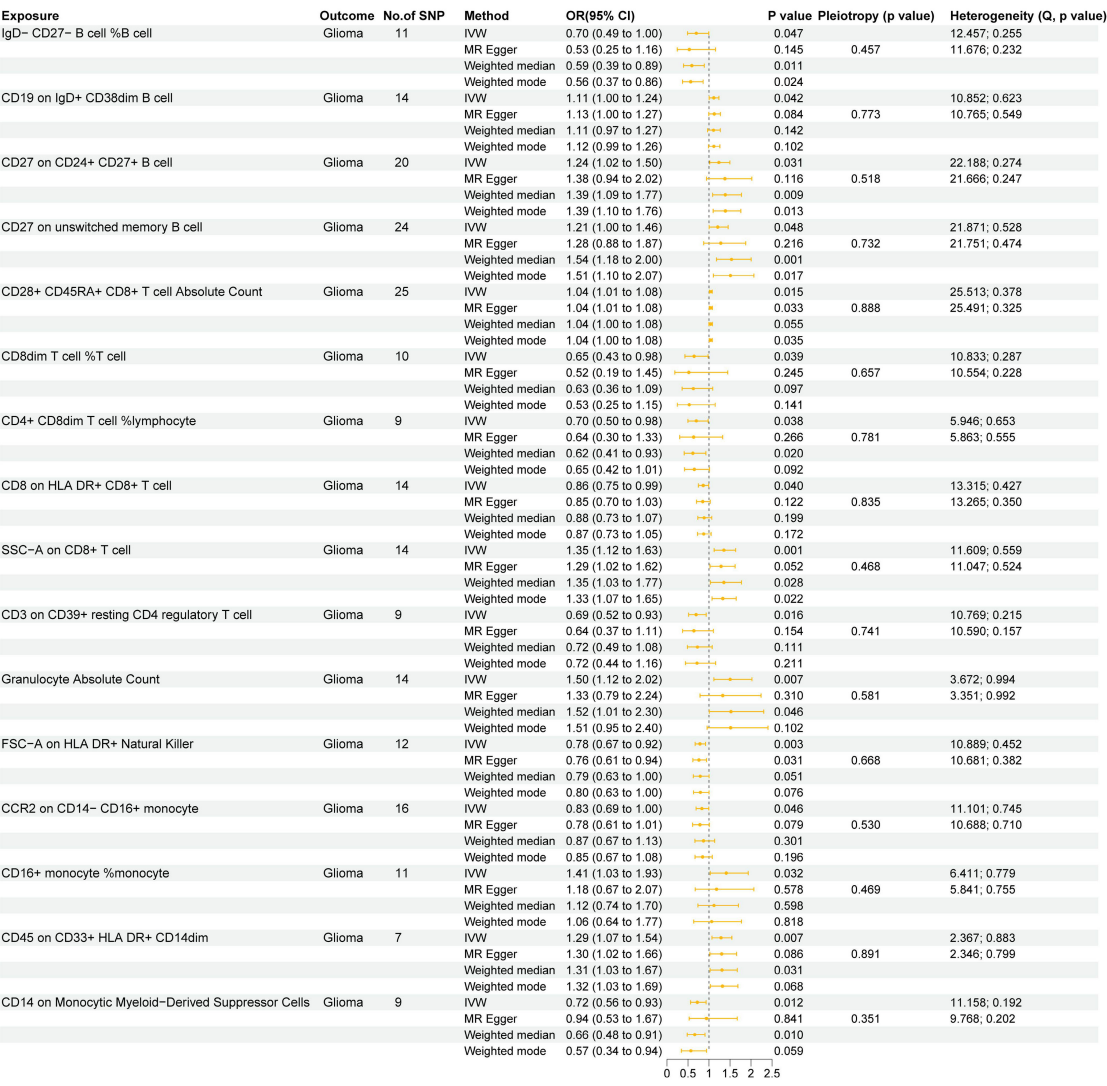
** Two sample MR results of causal effects of immune cell traits on glioma.** Data are expressed as an odds ratio (OR) with corresponding 95% confidence interval (CI). The forest plot also includes the results of sensitivity analyses. * P < 0.05, ** P < 0.01.

**Figure 2:**
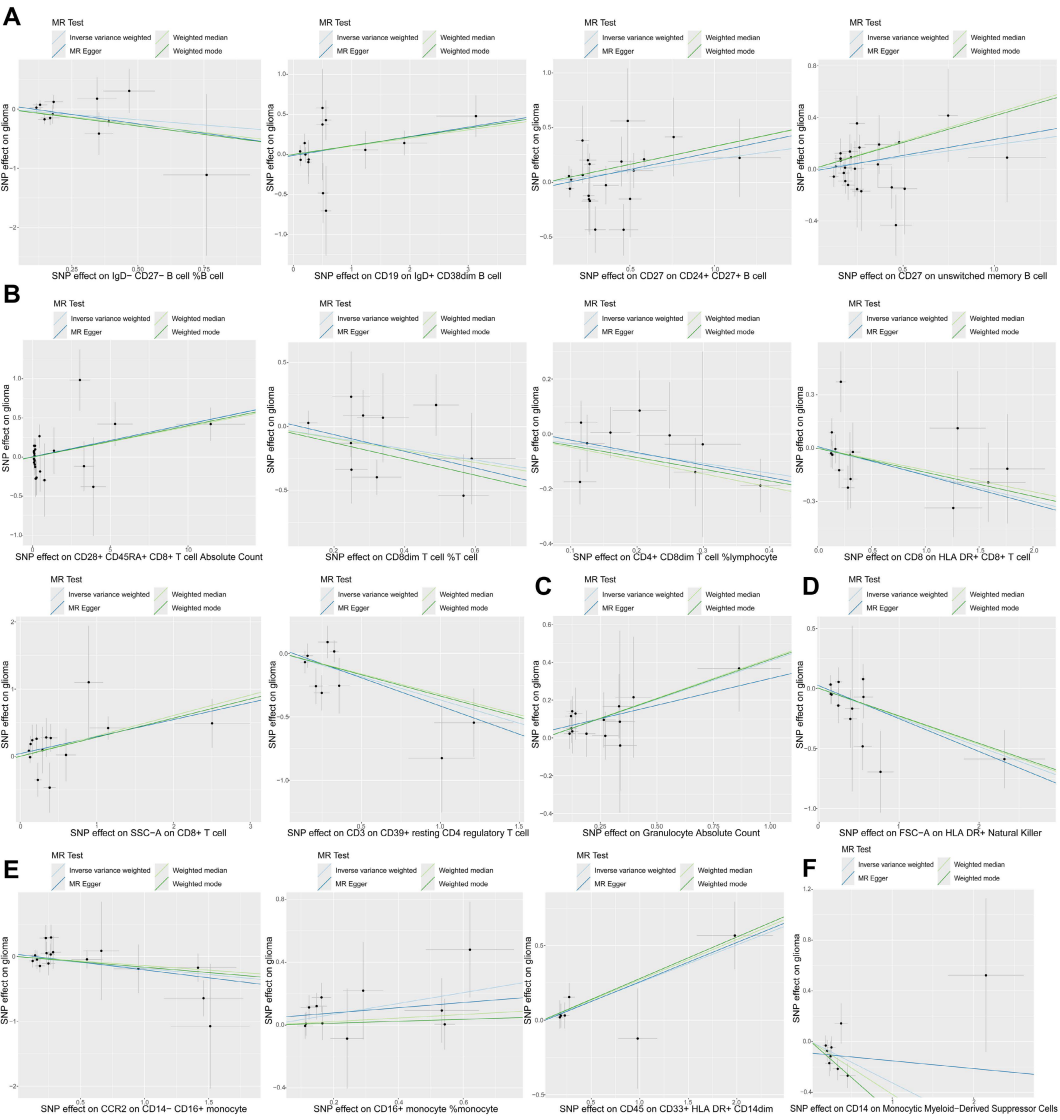
**The scatter plots of causal effects of immune cell traits on glioma. (A)** Potential causal effects of four B cell traits on glioma. **(B)** Potential causal effects of six T cell traits on glioma.** (C)** Potential causal effect of granulocyte absolute count on glioma.** (D)** Potential causal effect of FSC-A on HLA DR+ natural killer cells on glioma.** (E)** Potential causal effects of three monocyte traits on glioma.** (F)** Potential causal effect of CD14 on monocytic myeloid-derived suppressor cells on glioma.

In this study, we used two-sample MR analysis to explore the causal effects of 731 immune cell traits on the risk of glioma. The SNPs used for each immune cell trait were summarized in Supplementary Table S3, and the F statistics of theses SNPs ranged from 19.62 to 2435.82, with an average of 41.26, indicating that these SNPs were strong IVs. The results of MR analysis of all immune cell traits on glioma were summarized in Supplementary Table S4. Figure 1 summarizes the causal effects of 16 immune cell traits on glioma risk based on IVW analysis. Among these, four B cell traits showed significant associations: IgD- CD27- B cell %B cell (OR = 0.70, 95%CI = 0.49~1.00, p = 0.047) was negatively correlated with glioma risk, while CD19 on IgD+ CD38dim B cell (OR = 1.11, 95%CI = 1.00~1.24, p = 0.042), CD27 on CD24+ CD27+ B cell (OR = 1.24, 95%CI = 1.02~1.50, p = 0.031), and CD27 on unswitched memory B cell (OR = 1.21, 95%CI = 1.00~1.46, p = 0.048) were positively correlated with glioma risk. In addition, IVW analysis showed that six T cell traits were associated with glioma, including CD8dim T cell %T cell (OR = 0.70, 95%CI = 0.49~1.00, *p* = 0.047), CD4+ CD8dim T cell%lymphocyte (OR = 0.70, 95%CI = 0.50~0.98, *p* = 0.038), CD8 on HLA DR+ CD8+ T cell (OR = 0.86, 95%CI = 0.75~0.99, *p* = 0.040) and CD3 on CD39+ resting CD4 regulatory T cell (Tregs) (OR = 0.69, 95%CI = 0.52~0.93, *p* = 0.016), which were negatively correlated with the risk of glioma, and CD28+ CD45RA+ CD8+ T cell Absolute Count (OR = 1.04, 95%CI = 1.01~1.08, *p* = 0.015) and SSC-A on CD8+T cell (OR = 1.35, 95%CI = 1.23~1.63, *p* = 0.001), which were positively correlated with glioma. CD8+ T cells are critical components of the anti-tumor immune response, as they directly target and eliminate tumor cells through cytotoxic activity. The negative correlation between CD8 on HLA DR+ CD8+ T cells and glioma risk highlights the anti-tumor immune role of mature CD8+ T cells. In addition, IVW analysis also found that FSC-A on HLA DR+ Natural Killer (OR = 0.78, 95%CI = 0.67~0.92, *p* = 0.003), CCR2 on CD14-CD16+ monocyte (OR = 0.83, 95%CI = 0.69~1.00, *p* = 0.046) and CD14 on Monocytic MDSCs (OR = 0.72, 95%CI = 0.56~0.93, *p* = 0.012) were negatively correlated with the risk of glioma, and Granulocyte Absolute Count (OR = 1.50, 95%CI = 1.12~2.02, *p* = 0.007), CD16+ monocyte %monocyte (OR = 1.41, 95%CI = 1.03~1.93, *p* = 0.032) and CD45 on CD33+HLA DR+CD14dim (OR = 1.29, 95%CI = 1.07~1.54, *p* = 0.007) were positively correlated with the risk of glioma. Monocytes can differentiate into TAMs, which are known to promote tumor growth, angiogenesis, and immune suppression in the glioma microenvironment. The association of CD16+ monocyte %monocyte with glioma emphasizes the potential role of monocytes and their derivatives in the pathogenesis of glioma. The results of the other three MR analysis methods showed the same causal direction as the IVW analysis, supporting and supplementing the IVW analysis. Figure 2 presents scatter plots illustrating the genetic associations between the 16 immune cell traits and glioma risk. Each plot displays the SNP-exposure and SNP-outcome associations, with the slope of the regression line representing the causal effect estimated by MR analysis.

**Figure 3:**
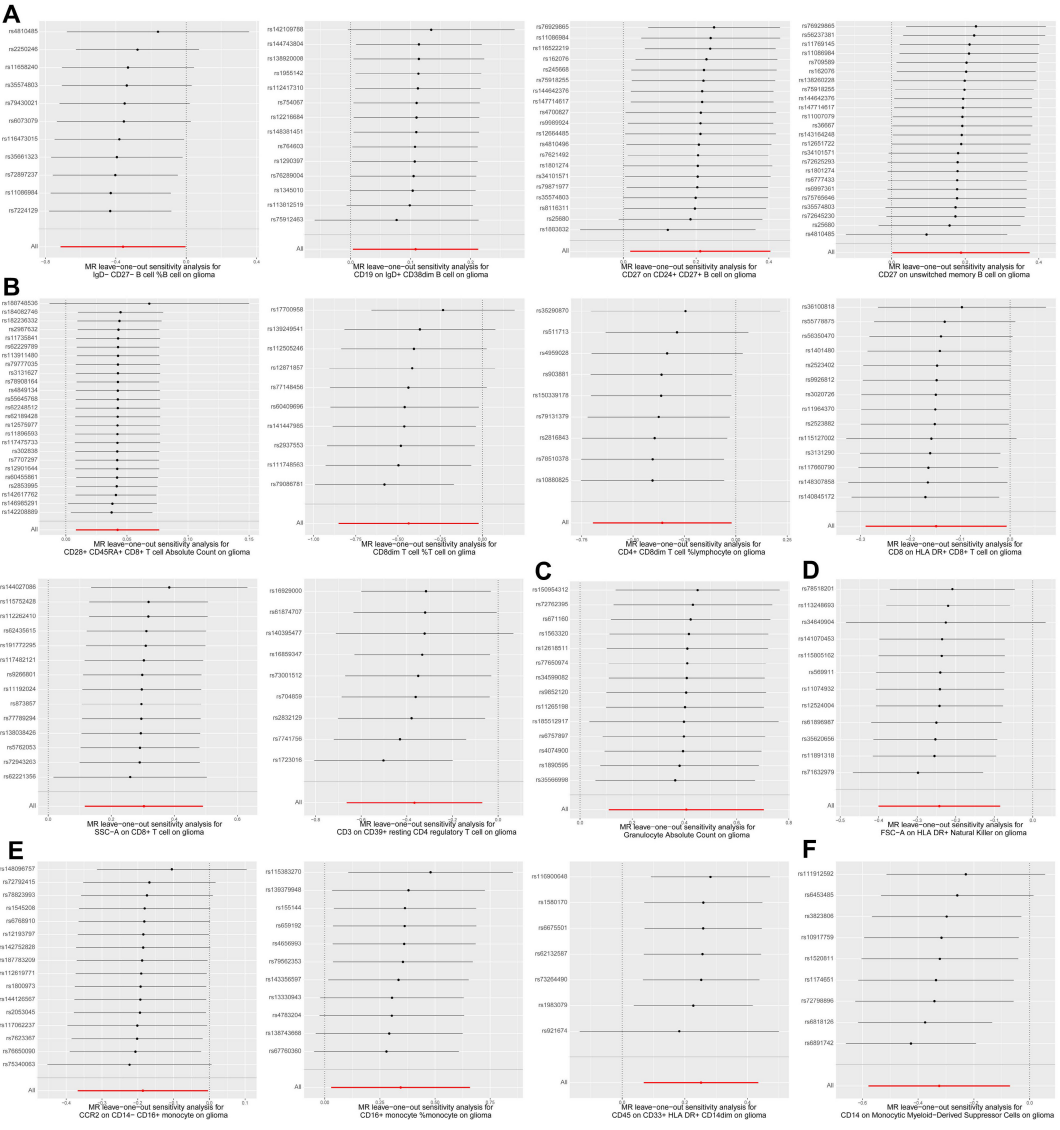
**Leave-one-out plots for two sample MR results of causal effects of immune cell traits on glioma. (A)** Leave-one-out plots of four B cell traits on glioma. **(B)** Leave-one-out plots of six T cell traits on glioma.** (C)** Leave-one-out plot of granulocyte absolute count on glioma.** (D)** Leave-one-out plot of FSC-A on HLA DR+ natural killer cells on glioma.** (E)** Leave-one-out plots of three monocyte traits on glioma.** (F)** Leave-one-out plot of CD14 on monocytic myeloid-derived suppressor cells on glioma. Forest plot of causal estimates omitting each variant in turn.

Next, we performed various sensitivity analyses on the MR analysis results between immune cell traits and glioma. The results of the horizontal pleiotropy analysis based on MR-Egger method were summarized in Supplementary Table S5, and the results of the heterogeneity analysis based on IVW and MR-Egger methods were summarized in Supplementary Table S6. As shown in Figure 1, we found no obvious horizontal pleiotropy or heterogeneity in the aforementioned 16 causal associations (all *p* > 0.05). Figure 3 shows the results of leave-one-out sensitivity analyses for the 16 immune cell traits. The consistent causal estimates across all analyses, with no single SNP driving the results, indicate the robustness of our findings and the absence of outlier SNPs. These results further support the complex and critical role of immune cells in the development of glioma.

### Immune cells in gliomas correlate with clinical characteristics

**Figure 4:**
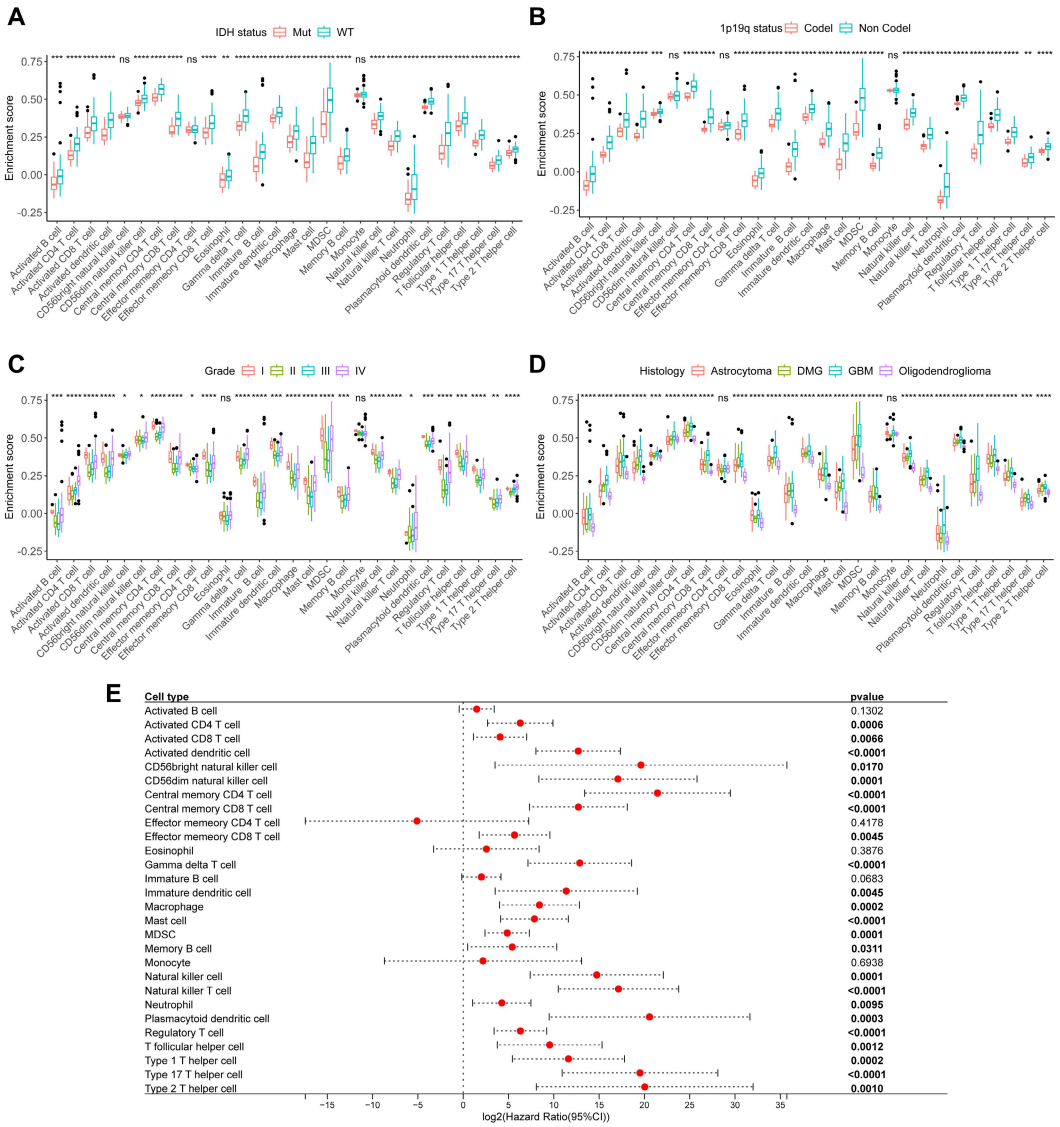
** The relationship between immune cells and clinical characteristics in CSUXY cohort. (A)** The abundance differences of immune cells between IDH mutant and IDH wild-type gliomas.** (B)** The abundance differences of immune cells between 1p19q codel and 1p19q non-codel gliomas. **(C)** The abundance differences of immune cells among different WHO grades. **(D)** The abundance differences of immune cells among different histological subtypes.** (E)** Univariate Cox regression analysis of overall survival for 28 immune cells in glioma. * *P* < 0.05, ** *P* < 0.01, *** *P* < 0.001, **** *P* < 0.0001.

To further explore the relationship between immune cells and tumor clinical characteristics in glioma, we collected 151 glioma samples and performed RNA-Seq to establish the CSUXY cohort. In this cohort, we used the ssGSEA method to infer the relative abundance of 28 immune cells in gliomas and found that almost all immune cells in IDH wild-type gliomas were more abundant than those in IDH mutant gliomas (Figure 4A), such as activated CD8+ T cells and macrophages. Similarly, the abundance of immune cells was higher in 1p19q non-codel gliomas than in 1p19q codel gliomas (Figure 4B). For different grades of glioma, we found that both grade I and IV gliomas had higher immune cell infiltration than grade II-III gliomas (Figure 4C). This observation may be attributed to the limited sample size of grade I gliomas (n=4) and requires validation in larger cohorts. It is possible that immune infiltration in grade I gliomas represents an early anti-tumor immune response aimed at controlling tumor growth at its initial stages. Activated CD8+ T cells, known for their cytotoxic activity, may infiltrate the tumor microenvironment to target neoplastic cells before the tumor develops advanced immune evasion mechanism.

**Figure 5:**
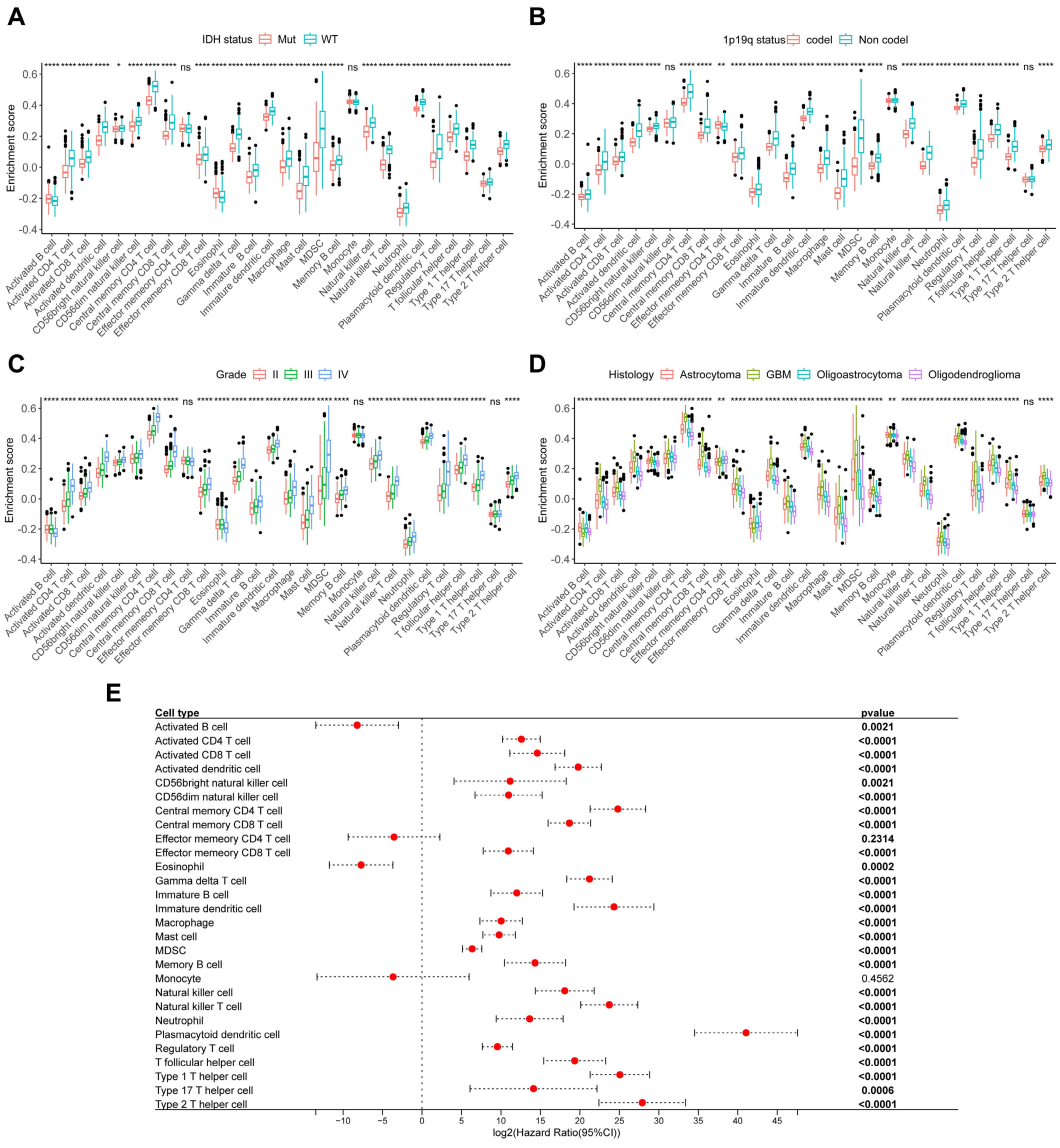
** The relationship between relative immune cells abundance and clinical characteristics in TCGA cohort. (A)** The abundance differences of immune cells between IDH mutant and IDH wild-type gliomas.** (B)** The abundance differences of immune cells between 1p19q codel and 1p19q non-codel gliomas. **(C)** The abundance differences of immune cells among different WHO grades. **(D)** The abundance differences of immune cells among different histological subtypes.** (E)** Univariate Cox regression analysis of overall survival for 28 immune cells in glioma. * *P* < 0.05, ** *P* < 0.01, *** *P* < 0.001, **** *P* < 0.0001.

**Figure 6:**
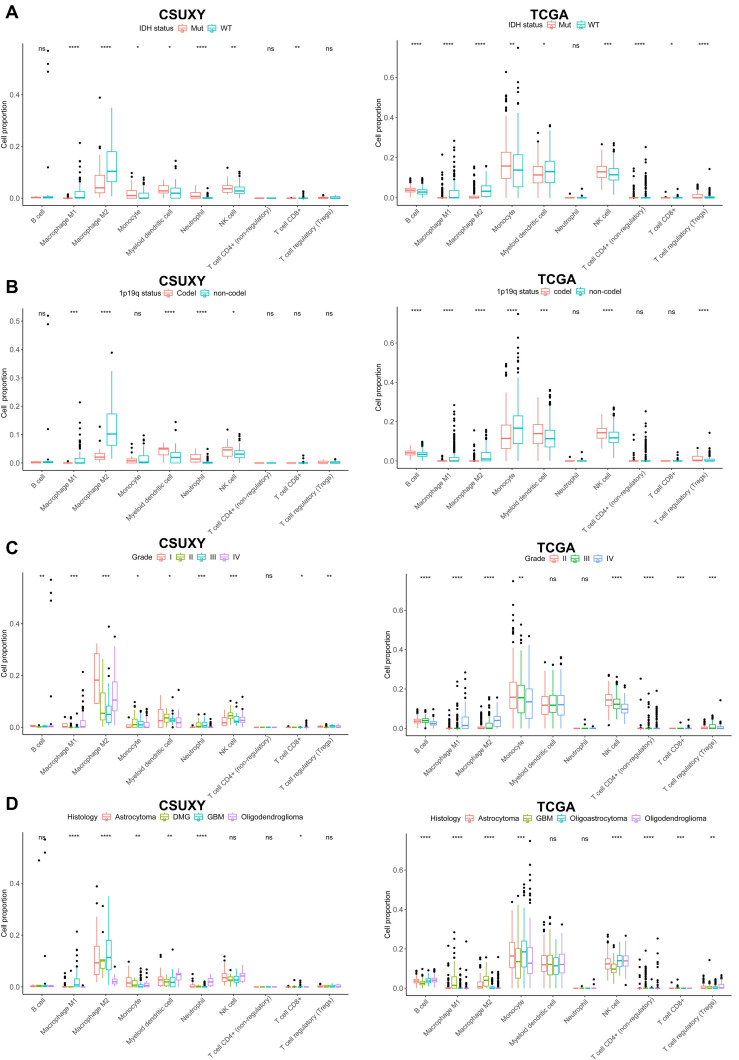
** The relationship between absolute immune cell proportion and clinical characteristics in CSUXY and TCGA cohorts. (A)** The proportion differences of immune cells between IDH mutant and IDH wild-type gliomas. **(B)** The proportion differences of immune cells between 1p19q codel and 1p19q non-codel gliomas. **(C)** The proportion differences of immune cells among different WHO grades.** (D)** The proportion differences of immune cells among different histological subtypes. * *P* < 0.05, ** *P* < 0.01, *** *P* < 0.001, **** *P* < 0.0001.

In addition, GBM had a higher abundance of immune cell infiltration compared to other histological subtypes of glioma, while oligodendroglioma had the lowest immune infiltration (Figure 4D). Univariate Cox analysis showed that the abundance of most immune cells was associated with poor prognosis in glioma (Figure 4E). We then used the TCGA cohort for validation analysis. Consistent with the CSUXY cohort, IDH wild-type and 1p19q non-codel gliomas had higher levels of immune cell infiltration than IDH mutant and 1p19q codel gliomas (Figure 5A-B). Grade IV gliomas had higher levels of immune infiltration than grade II-III gliomas (Figure 5C). In terms of histological subtypes, GBM also had the highest level of immune cell infiltration, while oligodendroglioma had the lowest level (Figure 5D). Interestingly, in the TCGA cohort, although the abundance of most immune cells was associated with poor prognosis in glioma, a higher abundance of activated B cells and eosinophils was associated with better prognosis in glioma (Figure 5E). Furthermore, we quantified the absolute proportions of 10 types of immune cells in both internal and external using the quanTIseq algorithm (Supplementary Table S7,S8). The immune cell composition of different samples in glioma has obvious heterogeneity (Supplementary Figure S1). Most immune cells in gliomas were myeloid-derived cells such as M2 macrophages, while the absolute proportion of T-cells is very low, indicating the dominant role of suppressive macrophages in the TME of glioma (Figure 6). This result is also consistent with previous studies 6,7. From a clinical perspective, in both the CSUXY and TCGA cohorts, the proportion of M2 macrophages was significantly higher in IDH wild-type and 1p19q non-codel gliomas compared to IDH mutant and 1p19q codel gliomas (Figure 6A,B). Conversely, the proportion of NK cells was significantly lower in IDH wild-type and 1p19q non-codel gliomas (Figure 6A,B). These findings suggest a predominance of immunosuppressive cells in IDH wild-type and 1p19q non-codel gliomas. Similarly, in both cohorts, the proportion of M2 macrophages was significantly higher in grade IV gliomas compared to grade II-III gliomas, while the opposite was true for NK cells (Figure 6C). Regarding the distribution of immune cells across different glioma subtypes, M2 macrophages were most abundant in GBM and least abundant in oligodendroglioma, whereas NK cells were least abundant in GBM and most abundant in oligodendroglioma (Figure 6D).

**Figure 7:**
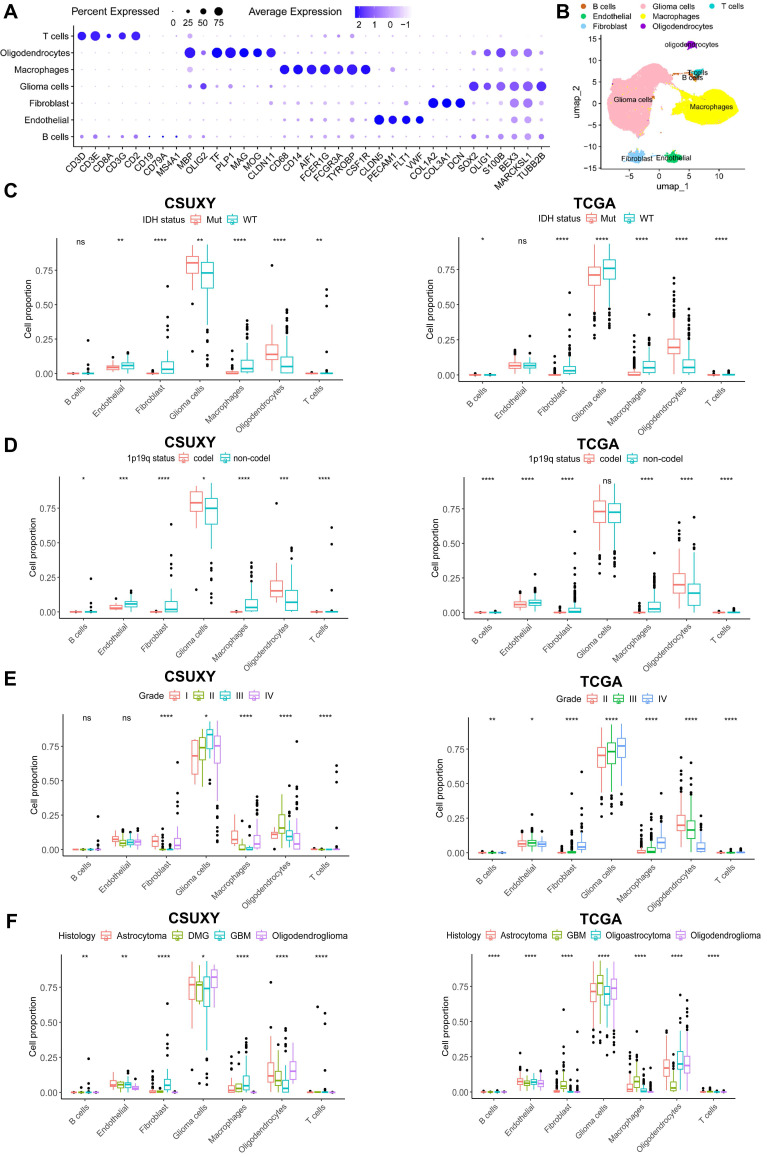
** The relationship between absolute immune cell proportion and clinical characteristics in CSUXY and TCGA cohorts based on scRNA-seq. (A)** The dot plot shows the expression of characteristic genes in seven cell types. The size of the dots indicates the proportion of cells expressing a specific marker, and the color indicates the average expression level of the markers.** (B)** The UMAP plot of the seven main cell types in glioma**. (C)** The proportion differences of immune cells between IDH mutant and IDH wild-type gliomas.** (D)** The proportion differences of immune cells between 1p19q codel and 1p19q non-codel gliomas. **(E)** The proportion differences of immune cells among different WHO grades. **(F)** The proportion differences of immune cells among different histological subtypes. * *P* < 0.05, ** *P* < 0.01, *** *P* < 0.001, **** *P* < 0.0001.

In addition, we performed scRNA-seq on nine glioma samples and identified seven major cell types based on the expression of signature genes (Figure 7A,B). We then performed deconvolution analysis on the CSUXY and TCGA cohorts based on the scRNA-seq results to infer the cell composition. Consistent with previous results, scRNA-seq-based analysis also showed that in addition to tumor cells and oligodendrocytes, the most common immune cell type was macrophages, while the absolute proportions of T cells and B cells were low (Figure 7B-F). In both the CSUXY and TCGA cohorts, IDH wild-type and 1p19q non-codel gliomas had higher proportions of macrophages, fibroblasts, and T cells compared with IDH mutant and 1p19q codel gliomas (Figure 7C,D). Regarding different grades of glioma, in both cohorts, the proportions of macrophages, fibroblasts, and T cells were significantly higher in grade IV gliomas than in grade II-III gliomas (Figure 7E). For different glioma subtypes, macrophages, fibroblasts, and T cells were most abundant in GBM and least abundant in oligodendrogliomas (Figure 7F).

### Immune cells in gliomas correlate with drug sensitivities

**Figure 8:**
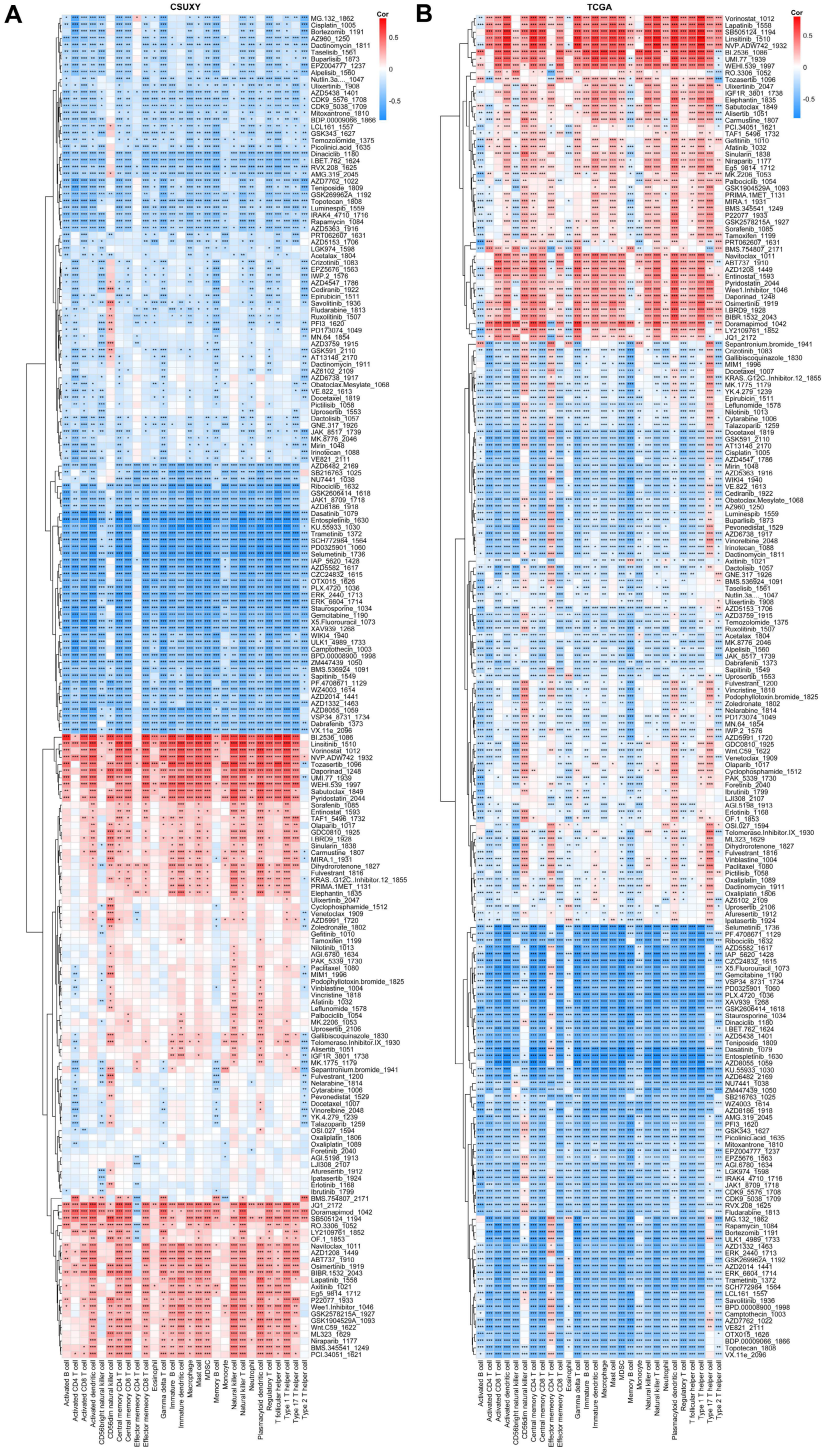
** Correlations between abundance of immune cells and drug sensitivities in CSUXY cohort (A) and TCGA cohort (B).** Correlation coefficients are calculated by Spearman's correlation analysis, with blue representing negative correlations and red representing positive correlations. * *P* < 0.05, ** *P* < 0.01, *** *P* < 0.001.

We have shown in a previous study that the TME status of tumors is associated with drug sensitivity 23, so we speculated that the infiltration abundance of specific immune cells may be associated with the drug sensitivity of gliomas. Based on the response data of 198 drugs from GDSC, we estimated the IC50 value of each sample in CSUXY and TCGA cohorts and then calculated the correlation of each drug's IC50 value with specific immune cells. Detailed information and targets of each drug were summarized in Supplementary Table S9. As shown in Figure 8A, in the CSUXY cohort, CD56dim natural killer cells were significantly positively correlated with the IC50 values ​​of RTK inhibitors such as Savolitinib and AZD3759, representing lower sensitivity to RTK inhibitors. Effector memory CD4+ T cells were specifically negatively correlated with the IC50 values ​​of BMS.754807, JQ1, and Doramapimod. Other immune cells were positively correlated with the IC50 values ​​of nearly half of drugs, and negatively correlated with the IC50 values ​​of the other half of drugs. In particular, most immune cells were strongly positively correlated with the IC50 values ​​of cell cycle or DNA damage-related drugs such as B1.2536, Linsitinib, and Pyridostatin. These results were replicated in the TCGA cohort (Figure 8B). The extensive associations observed between immune cells and drug IC50 values imply the promising utility of immune cells as potential biomarkers for predicting drug sensitivity in glioma.

## Discussion

Gliomas are notorious for their high malignancy and poor prognosis, necessitating further exploration of risk factors and biomarkers for gliomas 43. While the complex role of immune cells in the development and progression of gliomas has been established 38, further research is needed to determine whether immune cells are associated with glioma risk. Exploring the potential of immune cells as biomarkers for gliomas also holds significant translational significance. In this study, we analyzed 731 immune cell traits using MR method and identified 16 immune cell traits that may affect glioma susceptibility. Furthermore, through the analysis of internal and external transcriptomics cohorts, we examined the association between the abundance of 28 immune cells and the clinical characteristics of glioma, and explored the correlation between immune cells and drug sensitivity.

Our analysis revealed that specific immune cell traits, particularly certain B and T cell populations, are associated with glioma risk. B cells play a multifaceted and often underappreciated role in glioma, where they can infiltrate the tumor microenvironment and adopt regulatory functions. Recent studies suggest that infiltrating B cells can produce immunosuppressive cytokines such as IL-10 and TGF-β, as well as express immune checkpoint molecules like PD-L1, which contribute to the establishment of an immunosuppressive milieu that favors tumor progression 44. These B cells are often found in an immature state, resembling plasmablasts, and express markers characteristic of regulatory B cells (Bregs), indicating a potential blockade in their maturation process 44. In terms of B cells, we observed that CD27 on CD24+ CD27+ B cell and CD27 on unswitched memory B cell were positively correlated with glioma risk, suggesting that CD27 on B cell might play a role in tumor development. CD27 is a memory B-cell marker 45, and the CD27 expression has been used to distinguish between memory and naive B cells 46. The significance of CD27 expression on B cells in the context of tumors remains sparsely reported. However, recent studies suggest that memory B cells may play a crucial role in tumor immunity and are associated with the prognosis of various solid tumors 47, especially the stressed memory B cells which correlate with poorer prognosis in most tumors 48. Interestingly, in our internal cohort (CSUXY) consisting of 151 glioma samples, the abundance of memory B cells was significantly negatively correlated with the prognosis of glioma patients, and this negative correlation was also confirmed in the external cohort (TCGA). From a mechanistic perspective, in the context of glioma, memory B cells may contribute to tumor growth by promoting an immunosuppressive microenvironment through the secretion of cytokines such as IL-10, which can inhibit anti-tumor T cell responses 49,50. Additionally, the presence of CD27+ memory B cells may enhance the recruitment of Tregs and MDSCs, further dampening the immune response against the tumor 50-52. From the perspective of literature comparison, our findings are both consistent with and extend previous research on the role of immune cells in glioma. For instance, the association between memory B cells (CD27+ CD24+ B cells) and increased glioma risk aligns with recent studies suggesting that memory B cells may contribute to tumor progression by promoting an immunosuppressive microenvironment 50,53. Further investigation into the precise roles and mechanisms of B cells in gliomas is crucial for developing novel immunotherapeutic strategies. Such strategies may enhance antitumor immunity by modulating B-cell functions within the TME.

In terms of T cells, we discovered that the absolute count of CD28+CD45RA+CD8+ T cells and SSC-A on CD8+ T cells were also associated with an increased risk of glioma. Although current studies have demonstrated that tumor infiltration of CD28+ CD8+ T cells is associated with better prognosis in glioma 54, CD45RA, as a marker of naive T cells, indicates that CD28+ CD45RA+ CD8+ T cells possess characteristics of naive T cells. The presence of CD45RA on CD8+ T cells suggests that these cells may have a reduced capacity to proliferate and mount an effective anti-tumor response 55. Existing research has shown that pancreatic cancer patients with a lower proportion of peripheral naive T cells have a longer survival time 56. Furthermore, a study by Javier Carrasco et.al also revealed that CD45RA+ T cells lack proliferative capacity 57. The association between CD28+ CD45RA+ CD8+ T cells and increased glioma risk may be mediated through the exhaustion of naive T cells. SSC represents the inherent cell granularity of lymphocytes, which is closely related to cell function and state. The granularity of CD8+ T cells, as indicated by SSC-A, may reflect their functional state, with higher granularity associated with reduced proliferative capacity and impaired anti-tumor activity. Recent studies also have shown that CD8+ T cells with low SSC have a significantly higher proliferation rate than those with high SSC 58. CD28+CD45RA+CD8+ T cells and SSC-A on CD8+ T cells may increase the risk of glioma through lower anti-tumor immunity. These findings suggest that the immune landscape in glioma is shaped by a complex interplay of immune cell subsets, each contributing to tumor progression through distinct biological pathways. The negative correlation between CD8dim T cells and glioma risk is consistent with the well-established role of CD8+ T cells in anti-tumor immunity, where their reduced infiltration or functional exhaustion is often associated with poorer outcomes in various cancers, including glioma 59,60. These findings corroborate the importance of T cell-mediated immunity in controlling tumor growth and highlight the potential of targeting T cell exhaustion as a therapeutic strategy in glioma. However, some of our findings represent novel insights into the immune landscape of glioma. For example, the positive association between SSC-A on CD8+ T cells and glioma risk has not been previously reported. While previous studies have linked T cell granularity to proliferative capacity in other cancers 58, our study is the first to implicate this trait in glioma pathogenesis. T cells play a critical role in the immune response against gliomas, and their functionality varies significantly across different molecular subtypes of the tumor. In mesenchymal (MES)-like GBM, there is a higher infiltration of CD3+ and CD8+ T cells, which correlates with a more aggressive tumor phenotype and poorer prognosis. Conversely, the proneural subtype tends to exhibit a lower density of tumor-infiltrating lymphocytes, which may contribute to its relatively better clinical outcomes 61. Understanding these subtype-specific T cell dynamics is essential for developing tailored immunotherapeutic strategies that can enhance T cell responses and improve patient outcomes in glioma.

In this study, we collected 151 glioma samples with varying clinical characteristics for RNA-Seq analysis to explore the relationship between immune cell abundance and clinical features. The higher levels of immune infiltration observed in IDH wild-type and 1p19q non-codel gliomas suggest that these tumors may possess features that render them more immunogenic. This aligns with the hypothesis that certain genetic alterations can influence the immune landscape of tumors, potentially affecting treatment responses 62. Our findings indicated that gliomas of different grades exhibited varying levels of immune cell infiltration. Specifically, in both internal and external cohorts, high-grade gliomas tend to exhibit a higher degree of anti-tumor immune cell infiltration, such as activated CD8 T cells, compared to low-grade gliomas, but they also show a higher level of immunosuppressive cell infiltration, including Tregs and MDSCs. Consistent with this, the abundance of most immune cells is negatively correlated with the prognosis of gliomas, suggesting that the function of anti-tumor immune cells may be exhausted or suppressed in high-grade gliomas. Our results regarding the association between immune cell infiltration and glioma grade are consistent with previous studies showing that high-grade gliomas exhibit increased immune cell infiltration, particularly of immunosuppressive cell types such as Tregs and MDSCs 63-65. However, it also suggests another therapeutic opportunity. Although glioma patients (such as MES-like GBM) with higher levels of immune infiltration have poorer prognosis, they could also respond more positively to dendritic cell vaccination or checkpoint inhibitors (such as anti-CTLA4 ipilimumab, anti-PD1 nivolumab, and pembrolizumab) 65. This may be because pre-existing anti-tumor immunity is released as immunosuppression is lifted 38,65,66. However, it is worth noting that the hypothesis that MES-like GBM is more susceptible to immune checkpoint inhibition has not yet been supported by clinical data, and clinical trials related to this are ongoing 65, and continued research is needed to better understand the clinical significance of these findings. While grade IV gliomas demonstrated heightened immune infiltration compared to lower-grade tumors, the association of grade I gliomas with higher immune abundance was unexpected and warrants further investigation. The small sample size of grade I gliomas in our cohort may not provide a comprehensive view and should be expanded in future studies to better understand this relationship. Furthermore, the correlation between the abundance of immune cells and prognosis in glioma patients suggests the feasibility of predicting glioma prognosis based on the TME. This feasibility has been confirmed in numerous studies 23,67, indicating the potential of immune cells as biomarkers to facilitate individualized treatment decisions for glioma.

Using RNA-Seq data, we have also conducted an initial exploration of the association between immune cells and drug sensitivity. In both cohorts, we identified widespread associations between immune cell abundance and sensitivity to various drugs, particularly notable for the majority of immune cells being associated with cell cycle or DNA damage-related therapeutics. It is established that the inhibition of DNA damage repair can elevate cytoplasmic DNA, trigger cGAS to produce cGAMP, promote the generation of neoantigens in tumors, and ultimately induce immune cell proliferation and antitumor immunity 68-70. However, patients already exhibiting high levels of immune cell abundance may derive lesser benefit from the antitumor immunity induced by DNA damage. In summary, the extensive associations observed between immune cell traits and drug sensitivity in glioma patients open new avenues for personalized medicine. The correlation of specific immune populations with IC50 values for various drugs suggests that the immune landscape could serve as a predictive biomarker for treatment response.

Given the heterogeneity of gliomas and the complexity of the immune response, incorporating immune profiling into clinical practice could lead to more effective treatment strategies. Our findings suggest that specific immune cell traits, such as the abundance of memory B cells and CD8+ T cells, could serve as biomarkers to guide personalized treatment decisions. For instance, patients with high levels of CD8+ T cell infiltration may benefit from immune checkpoint inhibitors, such as anti-PD-1 or anti-CTLA-4 therapies, which have shown efficacy in other cancers with a similar immune profile 71,72. Conversely, patients with high levels of immunosuppressive cells, such as Tregs or MDSCs, may require therapies that target these cell populations, such as CSF1R inhibitors or IDO inhibitors, to enhance the efficacy of immunotherapies 73,74. In addition to guiding treatment selection, immune profiling could also be used to predict treatment response. For example, our observation that high immune infiltration is associated with poorer prognosis but better response to checkpoint inhibitors suggests that immune profiling could help identify patients who are most likely to benefit from these therapies. This is particularly relevant for high-grade gliomas, where the immune landscape is often more complex and heterogeneous 75. However, the TME in gliomas is characterized by complex interactions among various immune cell populations, including T cells, B cells, macrophages, and MDSCs. These interactions can significantly influence the immunogenicity of the tumor and the effectiveness of immunotherapy. For instance, while anti-PD-1 or anti-CTLA-4 therapy may be effective in patients with high levels of CD8+ T cell infiltration, the function of CD8+ T cells can also be impaired by the presence of immunosuppressive cells such as Tregs and MDSCs. These cells inhibit the activation and proliferation of T cells, thereby affecting the efficacy of immune checkpoint blockers 76. To optimize immunotherapy strategies, it is essential to adopt a holistic approach that considers the dynamic interactions among these immune cell types. This includes targeting not only the tumor cells but also the supportive immune cells within the TME. Strategies such as depleting MDSCs, reprogramming TAMs to a pro-inflammatory state, and enhancing the activation of CD8+ T cells can create a more favorable immune landscape that promotes effective anti-tumor responses 77,78. By comprehensively analyzing these immune interactions, it is possible to develop more effective and personalized immunotherapy approaches for glioma patients. For instance, the concurrent use of PD-1 inhibitors and CSF1R inhibitors targeting macrophages, combined with chemotherapy drugs, may hold promise. In addition, by incorporating immune profiling into routine clinical practice, clinicians could stratify patients based on their immune profiles and tailor treatment regimens accordingly, potentially improving survival outcomes. Furthermore, immune profiling could inform the development of novel immunotherapeutic strategies. For instance, the identification of specific immune cell subsets, such as CD16+ monocytes or SSC-A-high CD8+ T cells, as risk factors for glioma progression provides new targets for therapeutic intervention. Monoclonal antibodies or small molecule inhibitors targeting these cell populations could be developed to disrupt their pro-tumorigenic functions and enhance anti-tumor immunity. Furthermore, the association between infiltrating immune cells and circulating immune cells represents a critical aspect of tumor immunology. Studies have demonstrated dynamic interactions between these two populations. For instance, elevated levels of peripheral regulatory T cells correlate with increased tumor-infiltrating lymphocyte density 79, suggesting that systemic immune profiles may mirror the local tumor immune microenvironment. Consequently, investigating approaches to infer intratumoral immune status through peripheral blood could offer a promising non-invasive detection strategy.

Utilizing MR analysis and transcriptome data, this study explored the causal and clinical associations between immune cells and gliomas. Although our research offers valuable insights, there are admittedly some limitations. Despite our efforts to eliminate confounding factors, relying on GWAS summary data to investigate immune characteristics may still introduce potential biases related to population stratification and confounding variables, such as the inability to conduct stratified analysis based on gender and age. Furthermore, as the data in this study primarily originated from individuals of European descent, the ability to extrapolate the results to populations of other lineages is limited. Additionally, while the RNA-Seq analysis conducted on glioma samples is reliable, the cohort size needs to be further expanded, and the obtained results require confirmation in subsequent experiments. Future research should aim to validate these associations in larger and more diverse cohorts, and explore the underlying mechanisms that drive the relationship between immune cells and glioma progression.

## Conclusion

In summary, our study elucidates the causal relationship between immune cell traits and glioma risk, alongside their associations with clinical characteristics and drug sensitivity. These findings advance the development of immune-based biomarkers and therapeutic strategies for glioma, with potential implications for improving patient outcomes. Continued research in this area holds the promise of transforming glioma treatment through innovative, personalized approaches that leverage the body's immune response.

## Supplementary Material

Supplementary figure and tables.
