# Supplementary figures and images for: Exploring the Role of Immune Cells in Glioma: Causal Associations and Clinical Implications
Source: Int J Med Sci. 2025 Jun 12;22(12):2973–91. doi: 10.7150/ijms.116560 (PMC12244049; doi:10.7150/ijms.116560)

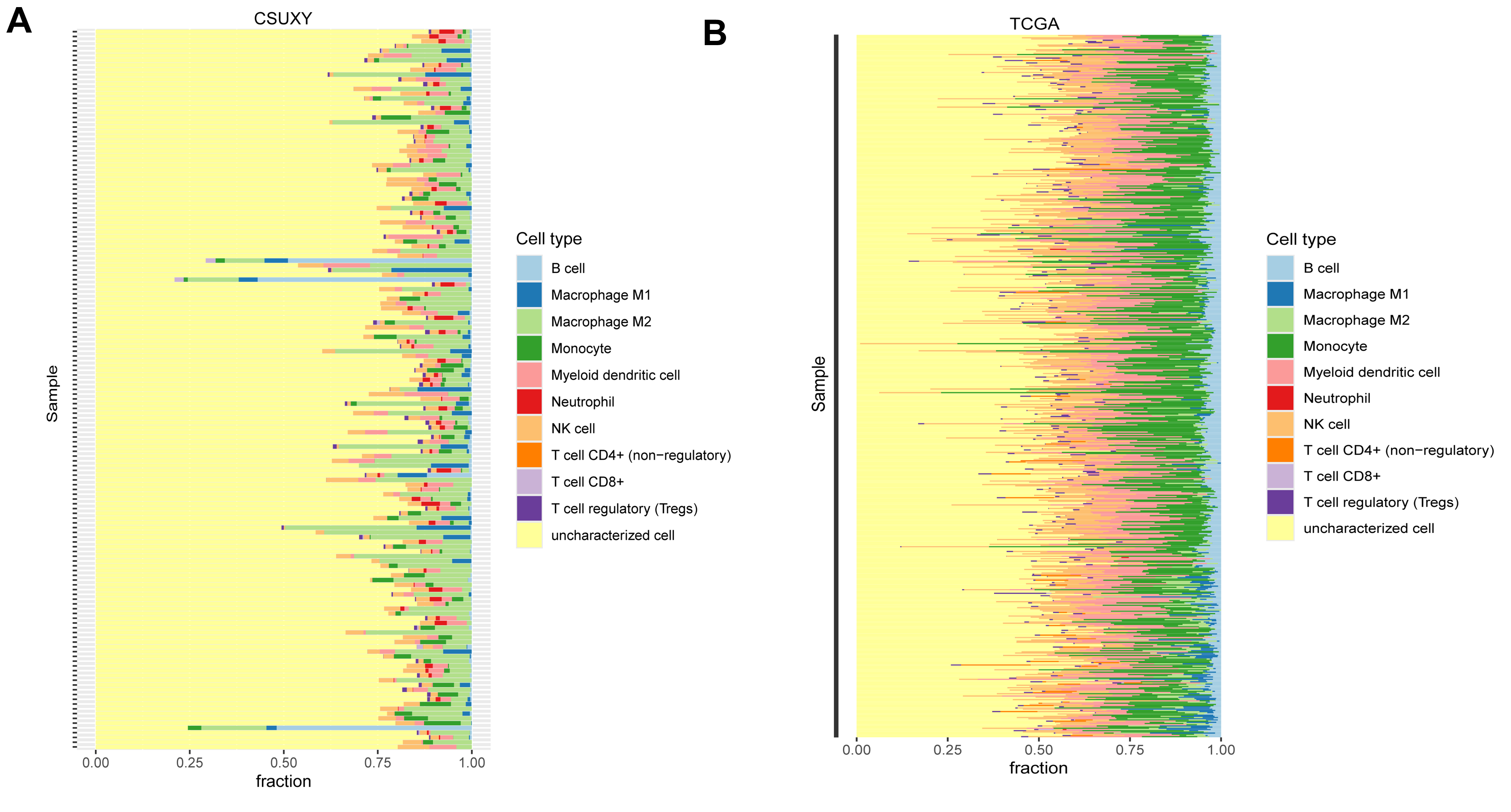

Supplement: Supplementary file 1 — Supplementary figure and tables. [file ijmsv22p2973s1.zip › Figure S1.tif]
